# Using rapid antigen testing for early, safe return-to-work for healthcare personnel after SARS-CoV-2 infection in a healthcare system in Pakistan: A retrospective cross-sectional study

**DOI:** 10.1371/journal.pgph.0001746

**Published:** 2023-03-22

**Authors:** Unab Inayat Khan, Syed Faisal Mahmood, Sara Khan, Zahra Hasan, Ahmed Cheema, Asif Hakim, Shehreen Inayat Ali

**Affiliations:** 1 Department of Family Medicine, Aga Khan University Medical College, Karachi, Pakistan; 2 Department of Medicine, Section of Infectious Diseases, Aga Khan University Medical College, Karachi, Pakistan; 3 Department of Medicine, Dean’s Research Fellow, Section of Infectious Diseases, Aga Khan University Medical College, Karachi, Pakistan; 4 Department of Pathology and Laboratory Medicine, Aga Khan University Medical College, Karachi, Pakistan; 5 Alumnus, Aga Khan University Medical College, Karachi, Pakistan; Federal University of Rio de Janeiro, BRAZIL

## Abstract

Anticipating staff shortage during the Omicron variant surge, we modified the US Centers for Disease Control and Prevention’s contingency guidelines at a healthcare system in Pakistan. Infected staff had a SARS-CoV-2 rapid antigen test after 5–7 days of isolation, to decide a safe return-to-work. This led to signifcant cost savings without compromising patient/staff safety.

## Introduction

In early 2022, the US Centers for Disease Control and Prevention (CDC) revised isolation guidelines for healthcare personnel (HCPs) with SARS-CoV-2 infection [1]. Balancing the risk of transmission to vulnerable patients and preventing staffing shortages [2], the new guidelines provide a two-tier isolation plan [3]. During times of high community burden of disease and anticipated staff shortage, contingency guidelines allow asymptomatic HCPs to return-to-work after a 5-day isolation (with or without repeat testing) [3].

During the COVID Omicron variant surge in Pakistan (January-March 2022), anticipating a workforce shortage, we implemented a modified CDC contingency plan at a private healthcare system in Pakistan. HCPs with PCR-confirmed SARS-CoV-2 infections had a rapid antigen test (RAT) after a 5-day isolation to decide on an early return-to-work.

Here, we describe our experience of determining the optimal duration of isolation (and safe return-to-work) using the rapid antigen test (RAT), and report the economic impact of implementing the modified CDC contingency plan as compared to the conventional 10-day isolation plan.

### Setting

The Aga Khan University’s healthcare system in Pakistan includes a tertiary care hospital, four secondary hospitals, 19 primary care sites, and 250 laboratory centers across the country. There are 13,960 employees with 80% involved in direct healthcare.

### Study design

We conducted a retrospective analysis on deidentified data of HCPs with PCR-confirmed SARS-CoV-2 infection obtained from the Office of Employee Health during January- March, 2022.

The study was approved by the AKU Ethics Review Committee (2020-5629-15118). As this was analysis of anonymized data, individual consent was not taken from HCPs.

### Testing policy

Assessment, testing and treatment were free for all HCPs through the Office of Employee Health, Department of Family Medicine, AKU.

All testing was performed at the Aga Khan University Hospital Clinical Laboratories, which are accredited by the College of American Pathologists, USA. SARS-CoV-2 infection was confirmed on a nasal specimen by polymerase chain reaction using the cobas SARS-CoV-2 Test by Roche Diagnostics [4]. Rapid Antigen testing (RAT) was conducted on nasal swabs using the LIAISON SARS-CoV-2 Ag assay, DiaSorin, Italy. The assay uses chemiluminescence immunoassay (CLIA) technology; and has a sensitivity of 95.4% (95%CI: 86.4–96.6%) and specificity of 100% (95%CI: 98.2–100%) [5].

The Employee Health COVID-19 database records information on all HCPs tested. It includes demographic information, area of work, the reason for testing, date of symptoms/exposure, date and result of testing, and dates and types of vaccination and booster doses against COVID-19.

### Modified return-to-work policy

We implemented a modified the return-to-work policy on January 15^th^ 2022 and reduced the isolation period from 10 days to 5–7 days. Between days 5–7 of positive SARS-CoV-2 PCR proven infection, an Employee Health nurse called the isolated HCP. If afebrile, a RAT was performed at the hospital’s testing facility. HCPs with a negative RAT were allowed to return to work the next day (day 6–8 of infection). HCPs who tested positive were instructed to complete a 10-day isolation and return on day 11 without further testing.

### Analysis

Categorical data are reported as frequencies and percentages, and normally distributed continuous variables as mean ± standard deviation. We used Pearson’s correlation coefficient to examine the correlation between percentage positivity and days of symptom onset and days of isolation after PCR test. A p-value of <0.05 was considered significant. The number of days saved was calculated by subtracting the actual return-to-work day from 11 (conventional return-to-work after 10-day isolation). Cost implications were calculated using Human Resources data. Analyses were completed using Stata version 15.

## Results

During the Omicron surge, 2035 HCPs were diagnosed with a PCR-confirmed COVID-19 infection. Of these, 1340 (70%) had a RAT to decide on early return-to-work. Comparing those who had an RAT and those who did not, we found no difference in age, area of work and vaccination status; while a higher proportion of female HCPs had an RAT compared to male HCPs (50.75% vs 49.25%; p = 0.005).

Amongst the 1340 infected HCPs who had a RAT, 1216 (90.7%) had symptomatic COVID-19 infection, where as 124 (9.3%) had asymptomatic COVID-19 infection. The mean age was 33.9 (± 8.83) years; and 680 (51%) were females. Of these, 908 (68%) were working in clinical non-COVID areas; 33 (2%) in clinical COVID areas; and 399 (30%) were working in non-clinical areas. Almost all had completed their primary vaccination series (99%) and 84% had received an mRNA vaccine booster. There were no differences in age, sex distribution, area of work between those who had a positive RAT and those who did not.

**Fig 1:**
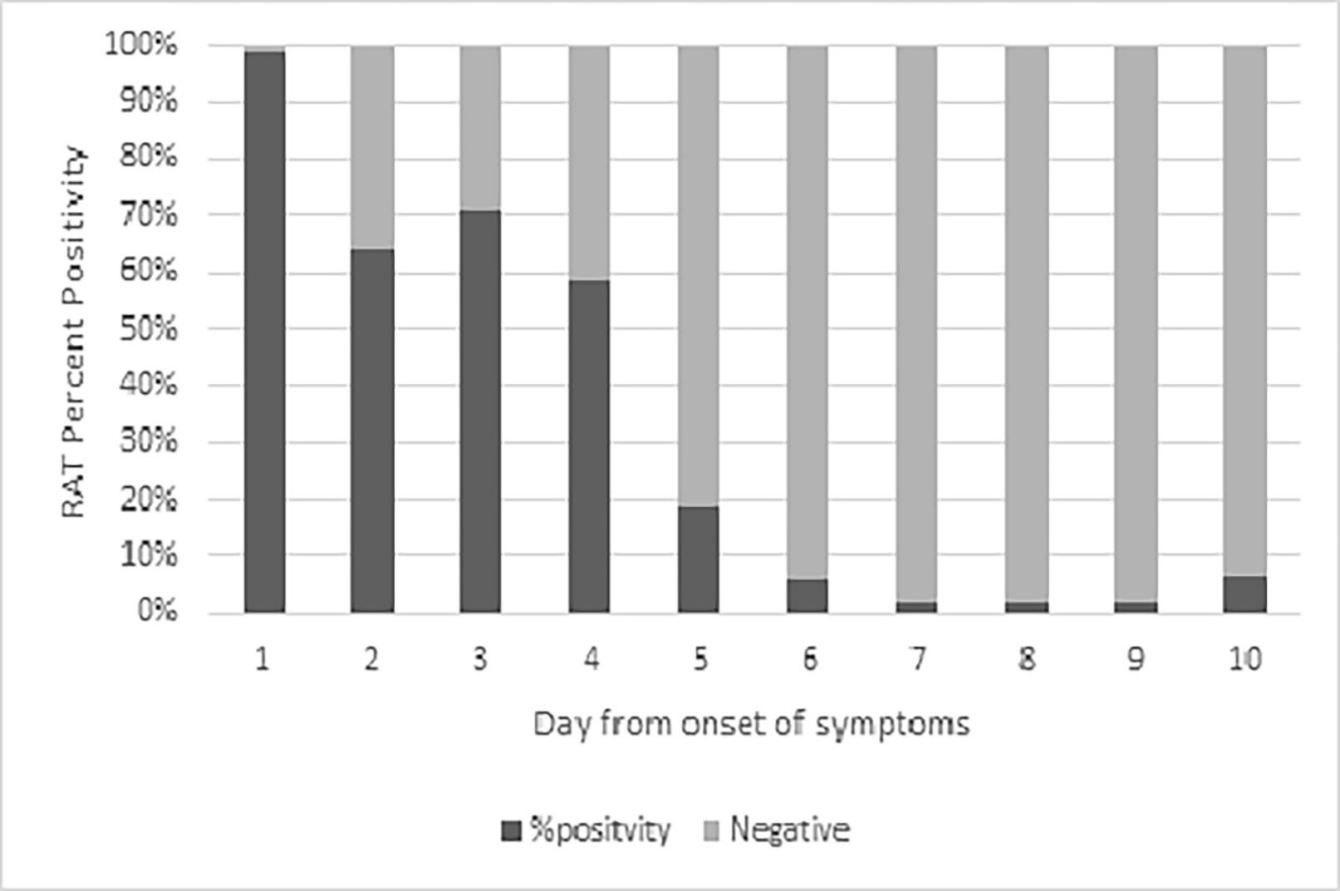
Percent positivity from day of symptom onset 1216 HCPs who had rapid antigen test (RAT) for early return-to-work.

As the new return-to-work policy was rolled out, the time between the first PCR and the follow-up RAT was not uniform, we were able to analyze the percent positivity of the RAT by day of symptoms. Fig 1 shows the percent positivity from the day of symptoms in the 1216 symptomatic HCPs. We see that the percent positivity was highest on day 1 where 2/3 (66%) were positive. On day 5, 27 of 123 (22%) HCPs tested positive, while on day 7 of symptoms, only 21 of 327 (6%) tested positive.

Percent positivity of RAT negatively correlated with days from PCR-test (r = -0.21; p <0.001) and days of symptom onset (r = -0.17; p <0.001). There was no correlation between percent positivity and age or vaccination status.

Table 1 shows the number of days saved by early return-to-work. Of the 1340 RATs conducted, 118 (8.8%) had a positive RAT at different times of testing, and had to complete a 10-day isolation; and 28 HCPs were tested on or after day 10 and would have returned to work without RAT with the conventional guidelines. Of the 1090 RATs done between days 5–9, we subtracted the day of return after a negative test, from 11 (return day after conventional 10-day isolation). This led to early return-to-work for 1022 HCPs and a savings of 3799 work-days. One full-time equivalent (FTE) works 300 days per year, this translates to 12.66 FTEs. For 12.66 registered nurses (average annual salary including benefits: USD 4216), this led to a cost-savings of USD 53,374. Considering the additional cost of USD 8657 for RATs (cost per RAT is USD 6.5), the modified protocol led to a direct net savings of USD 44,717 (PKR: 6,707,634 using the 2019 average conversion rate of 1.0 USD = 150 PKR) for the institution.

**Table 1:** Calculation of early return-to-work by day of negative RAT.

No. of HCPs with early return to work = 1022	Days saved*
Day 5 test: 266	5 * 266 = 1330
Day 6 test: 367	4 * 367 = 1468
Day 7 test: 269	3 * 269 = 807
Day 8 test: 74	2 * 74 = 148
Day 9 test: 46	1 * 46 = 46
Total days saved	3799

## Discussion

The CDC’s contingency plan for HCPs’ isolation and return-to-work at times of high community transmission bridges the gap between patient/HCP safety, mitigating spread and ensuring a functioning workforce. Our study examines a database of healthcare personnel with COVID-19 infection at a large healthcare system in Paksitan.

Shortening the isolation period could lead to potentially infectious HCPs returning to work, risking infections in vulnerable patients. Adding RAT to the early return-to-work policy may mitigate this concern. Viral load (and hence infectivity) peaks within the first 5 days of infection [6]; and RAT positivity is based on a higher viral threshold [7]. A positive RAT has been correlated with high infectivity, especially for the Delta [8] and Omicron variants [9]. Our study shows 22% of infected HCPs had a positive RAT on day 5; and the percent positivity drops to 6% by day 7. However, 1/14 HCP was still positive on day 10, showing that infectivity may be longer with the Omicron variant infection. Similar results have been noted by Lefferts et al, who report a 38% RAT-positivity on day 9 of a PCR confirmed infection during the Omicron surge in Alaska [10]. To find a pragmatic balance between safety and staff shortage, it may help to extend the return-to-work from day 5 to day 7; with the caveat that HCPs continue strict masking till day 10 or complete resolution of symptoms (whichever comes later).

We also show substantial institutional direct cost-savings despite the added cost of RAT. This becomes especially important in resource-constrained settings where trained HCPs are already limited. And this does not include the additional cost-savings of patient care as well as the decrease in work-burden on other HCPs.

Our study has certain limitations. It is possible that some HCPs presented later in the disease, resulting in incorrect RAT percent positivity calculation. Viral load peak is associated with severity of illness [11]. As most HCPs in our study had mild symptoms, and none required hospitalization, our results may not be valid for those with moderate to severe illness.

Despite these limitations, our findings do have wide applicability, especially in the setting of a surge, where HCP shortage is expected. And show the economic impact of early return-to-work despite the added cost of RAT.

## Supporting information

S1 Data Anonymized data. (DTA)
